# The Institutional Library

**Published:** 1914-12-12

**Authors:** 


### THE INSTITUTIONAL LIBRARY.

### French Practice of Urgent Surgery.

Urgent Surgery. By Félix Lejars. Translated from the Seventh French Edition by Wm. S. Dickie, F.R.C.S. Third English Impression. With twenty full-page plates and 1,086 illustrations. Volume I. (Bristol: John Wright and Sons, Ltd. 1914. Two Vols. Price 25s. each.)

Urgent Surgery is an expression which, at the present time, suggests war surgery; but in truth urgent surgery covers a wider field than that of bullet and shell wounds, and on the other hand war surgery is far from being always urgent. The wide range of urgent surgery is impressed upon the mind by the size and the scope of the two considerable volumes which Professor Félix Lejars has so successfully conducted through seven editions. It is now some fifteen years since the first edition of this standard French work appeared, and at that time the field included in the term "urgent surgery" was far less extensive than it is now. Professor Lejars has justly been regarded as the pioneer in this development of surgical work, and surgeons of all countries have appreciated the value of his teaching. But not alone on account of his operative acumen has Professor Lejars proved himself entitled to the gratitude of the surgical world; he has also the rarer gift of imparting in the clearest and most graphic manner a knowledge of surgical technique and treatment. A glance through his book will confirm this. Apart from the abundance of the illustrations, which show exceptionally clearly and realistically the important details of the several stages of the operations, there is to be found an excellence of description, both of minor and major points, which has rarely been surpassed in surgical text-books. The author does not disdain to describe adequately such simple matters as the way to hold a basin correctly and how to clean the nails. In the introductory chapter there is given an account of the equipment of the surgeon, which forms a useful guide for country practitioners, and the author describes at length in a few vivid pages how the surgeon should proceed in a case of urgency when necessaries are completely lacking. The impression gained from these pages is strengthened, as one peruses further, that the author is thoroughly practical, and is retailing the methods which he has found best as the result of long and often stern experience. One sentence deserves to be cited. After describing the preparations required in unfavourable surroundings Professor Lejars says, "Any time you may think to gain by unduly curtailing these preliminaries is time irretrievably lost. It is the actual operation which requires to be carried out quickly." Then, again, how necessary is the strict insistence on the proper cleansing of instruments after an operation; how often does the occasional operator in the country neglect this task, to his great discomfort on the next urgent occasion! If carefully and adequately done, this task requires time and trouble, but, as the author aptly states, "I know of others, willingly undertaken in the name of sport, which are less useful and far more troublesome."

The translation of the present edition, which is the seventh in French, forms the second English edition. Mr. W. S. Dickie, F.R.C.S., who translated the sixth French edition some five years ago, is again responsible for the English. His former success has been repeated, and he has produced not merely a translation, but reproduced something of the French spirit and atmosphere. There is no attempt at editing, and English surgeons will be interested in studying any points in which French technique and practice differ from their own. Owing to the greatly increased intercourse between surgeons of different nationalities, pure national characteristics tend to disappear. This book was noticed in our columns on its first appearance in its English garb, and has now such an established reputation that a detailed survey is uncalled for.

Practical Bandaging, including Adhesive and Plaster Dressings. By E. L. Eliason, A.B., M.D. (Philadelphia and London: J. B. Lippincott Company. 1914. Price 6s. net.)

A knowledge of bandaging may, at the present time, be reckoned as almost a fashionable failing. A smattering of the technique has been fairly widely disseminated among enthusiastic voluntary helpers at first-aid classes since the war began, but nurses and medical men know that the art of bandaging is by no means easy to acquire thoroughly, and that to become a practically useful and reliable bandager necessitates a long and attentive apprenticeship. If very realistic photographs and a profusion of diagrams mean half the battle being won, then Dr. Eliason's new text-book should find favour. As regards the technique of roller bandaging the book gives clear explanations, but it cannot be said that this American manual is in any way superior to those obtainable here and written by English doctors or matrons. Some of the illustrations are objectionable in their unnecessary realism. It is curious to find that the author does not draw attention to the point usually insisted upon by our surgeons, that a leg should be bandaged from within outwards; in fact, the diagrams show just the opposite. Manipulations with adhesive plasters claim a considerable space. The preparation and application of plaster-of-Paris casts are adequately described with the inclusion of several practical hints, but it cannot be said that this subject is treated exhaustively. Although a few words are devoted to silicate and to starch casings, no mention is made of celluloid splints. The chapter on miscellaneous bandages, such as handkerchiefs, triangulars, slings, etc., is useful and practical.

### A Practical Book for Residents.

Tuberculosis of the Bones and Joints in Children. By John Fraser, M.D., F.R.C.S.E. Illustrated with 51 full-page plates and 164 figures. (London: A. and C. Black. 1914. Edinburgh Medical Series. Price 15s.)

A book such as this should find a place on the bookshelves of many an institutional library; residents in general hospitals and in children's hospitals particularly would often find reference to its practical pages a help in dealing with cases of Pott's disease or in wrestling with Thomas's splints. No one, unless he be constantly engaged in treating cases of tuberculosis of the bones and joints, can afford to scorn the "looking up" of the numerous and important details, attention to which makes all the difference in the handling of these patients. In truth specialism is justified in the treatment of these very chronic and often very difficult cases. A study of the pages of Mr. Fraser's somewhat formidable manual will soon convince any house surgeon of the need for something more than mere routine treatment if these unfortunate children are to be relieved of their sufferings and deformities. In this admirably arranged text-book the author has been to some pains to treat the whole subject systematically and in due order, so that reference to any particular section is easy and sufficiently complete by itself to be practically useful. The book falls into two main divisions: general and special; the former accounts for about one-third of the pages, and is lavishly illustrated with many really beautiful plates depicting the microscopic anatomy of bone, subperiosteal bone formation and the histology of bone tubercle. The pathological and etiological aspects of the subject are fully gone into, and this part represents a great deal of personal investigation on the part of the author which was undertaken under Mr. Stiles, to whom, indeed, the book is dedicated. An admirable feature, not only of this section in which divergent views have to be met, but also throughout the book, is the extensive nature of the bibliography. These comprehensive lists add value to the work from a consultative point of view. The clinical features, diagnosis and treatment of both bone and joint tuberculosis, are dealt with separately in a manner which emphasises the salient points both for the student and the practitioner, and bears the hall-mark of practical experience. The subject of treatment is necessarily a complex one, but the author manages to accord to each of the branches of this very important section a full measure of attention; the methods of dealing with sinuses and cold abscesses are well described. Plaster-of-Paris fixation is particularly well written up, and all the minor, yet extremely important details connected with the manipulation of plaster bandages and the making of casts are properly emphasised. Tuberculin has also its place in the scheme of treatment, and as regards this the author is content to be cautious, and gives due weight to the uncertainties of this vexed question. There is, by the way, an error in the description of the reagent used for the Von Pirquet test (page 52), and this paragraph is scarcely an adequate presentation of the reaction. In the second part of the book we have descriptions of the disease as it affects individual regions, and here the author is at his best. The section on tuberculous disease of the spine is almost a masterpiece of completeness, and the details given regarding the making and fitting of jackets, braces, and other supports should satisfy the most captious, while the pathology of the disease is admirably investigated. The disease as it affects other joints and bones in children is everywhere illustrated thoroughly and clearly. The book deserves an established place among the really practical text-books of medicine and surgery, and is not likely to be the least appreciated volume of the excellent Edinburgh Medical Series now being prepared under the editorship of Dr. John Comrie.

